# Supplementary material for: Serological evidence for the presence of wobbly possum disease virus in Australia
Source: PLoS One. 2020 Aug 4;15(8):e0237091. doi: 10.1371/journal.pone.0237091 (PMC7402471; doi:10.1371/journal.pone.0237091)
Supplement: S1 Table — (DOCX) [file pone.0237091.s001.docx]

|  | WPDV seropositive, incl. equivocals | Seroprev., % | 95% CI | p value* |
| --- | --- | --- | --- | --- |
| **Species** |  |  |  | **0.001** |
| Common ringtail | 6/57 | 10.5 | 4.0 – 21.5 |  |
| Mountain brushtail | 9/31 | 29.0 | 14.2 – 48.0 |  |
| Common brushtail | 37/100 | 37.0 | 27.6 – 47.2 |  |
| **Sex** |  |  |  | 1.00 |
| Male | 30/106 | 28.3 | 20.0 – 37.9 |  |
| Female | 21/75 | 28.0 | 18.2 – 39.6 |  |
| Unknown | 1/7 | 14.3 | 0.3 – 57.9 |  |
| **Age** |  |  |  | **0.04** |
| Juvenile | 5/32 | 15.6 | 5.3 – 32.8 |  |
| Subadult | 4/26 | 15.4 | 4.4 – 34.9 |  |
| Adult | 43/126 | 34.1 | 25.9 – 43.1 |  |
| Unknown | 0/4 | 0.0 | 0.0 – 60.0 |  |
| **Environment** |  |  |  | 0.36 |
| Rural | 9/32 | 28.1 | 13.7 – 46.7 |  |
| Urban | 29/91 | 31.9 | 22.5 – 42.5 |  |
| Semi-urban | 14/65 | 21.4 | 12.3 – 33.5 |  |

*calculated using Fisher’s exact test, excluding the unknown groups
