# Supplementary material for: Serological evidence for the presence of wobbly possum disease virus in Australia
Source: PLoS One. 2020 Aug 4;15(8):e0237091. doi: 10.1371/journal.pone.0237091 (PMC7402471; doi:10.1371/journal.pone.0237091)
Supplement: Attachment — Submitted filename: Response to Reviewers.docx [file pone.0237091.s002.docx]

**Response to reviewers’ comments**

**Journal Requirements:**

1. *We note that [Figure 2] in your submission contain [map/satellite] images which may be copyrighted. All PLOS content is published under the Creative Commons Attribution License (CC BY 4.0), which means that the manuscript, images, and Supporting Information files will be freely available online, and any third party is permitted to access, download, copy, distribute, and use these materials in any way, even commercially, with proper attribution. For these reasons, we cannot publish previously copyrighted maps or satellite images created using proprietary data, such as Google software (Google Maps, Street View, and Earth). For more information, see our copyright guidelines:* [*http://journals.plos.org/plosone/s/licenses-and-copyright*](https://protect-au.mimecast.com/s/aM3bCL7rxDsRPjWONuruhpa?domain=journals.plos.org)*.* *We require you to either (1) present written permission from the copyright holder to publish these figures specifically under the CC BY 4.0 license, or (2) remove the figures from your submission. Please upload the completed Content Permission Form or other proof of granted permissions as an "Other" file with your submission. In the figure caption of the copyrighted figure, please include the following text: “Reprinted from [ref] under a CC BY license, with permission from [name of publisher], original copyright [original copyright year].”*

Author response: The map in figure 2 was sourced and reprinted from GEODATA TOPO 250K Series 3 (Geoscience Australia; http://pid.geoscience.gov.au/dataset/ga/64058), which is available online under Creative Commons Attribution License 4.0, as declared in the figure caption.

**Reviewers' comments:**


**Reviewer #1:**

1. *The manuscript is concise, organised, and contributes to the knowledge of a long-standing health problem in the common brushtail possum, but is most relevant to an Australasian, or wildlife disease readership. The manuscript adds to our knowledgebase of the geographic distribution, host exposure, and incidence of the group of viruses associated with the wobbly possum disease syndrome. Although a very publishable manuscript, I would tend towards recommending a rejection while providing encouragement to submit to journals with a more targeted readership.*

Author response: Thank you for these comments and for the careful review. PLOS one publishes papers across a broad range of subject areas, including the natural sciences. We feel the paper is a good fit for the scope of the journal.

2. *The manuscript is technically sound and the data support the conclusions. Nonetheless, the authors make it clear that another paper has been published describing the disease syndrome in Australia (citation below). The findings of the recent publication on the topic have not incorporated in the submitted manuscript introduction and are not fully woven into the discussion. This approach leaves the reader quite isolated and not updated on the current breadth of knowledge on the disease syndrome either in the NZ nor the Australian context. A more detailed introduction summarising the similarities and differences between Australian and New Zealand wobbly possum disease, including concise descriptions of the temporal, geographic ranges, putative aetiologic agents identified and comparative pathology would ensure readers have a wholistic understanding of the context for the investigation described.

   Recent citation:
   Chang, W., Eden, J., Hartley, W.J. et al. Metagenomic discovery and co-infection of diverse wobbly possum disease viruses and a novel hepacivirus in Australian brushtail possums. One Health Outlook 1, 5 (2019).* [*https://doi.org/10.1186/s42522-019-0006-x*](https://doi.org/10.1186/s42522-019-0006-x)

Author response: At the time of submission this citation was a preprint only and so it was difficult to discuss the findings with confidence. As this has now been published (and the sequences of the Australian viruses have become available), we have expanded our manuscript to include more discussion of the findings. We have also expanded our introduction section to include some more information about the similarities and differences between Australian and New Zealand wobbly possum disease (see lines 38-72, page 3-4 in revised manuscript). In addition, we have included a multiple alignment of the predicted aa sequences of protein N from Australian and New Zealand viruses, as this protein was used as an antigen in the blocking ELISA test (page 7, lines 130-135).

3. *Line 46 – summarising the histopathology – should include choroid and optic tract, should indicate that the histological lesions vary regionally (CNS only on mainland Australia, and multisystemic LP inflammation in NZ and Tas)*

Author response: Additional information has been added to the revised manuscript (see line 51-56), incorporating the pathology associated with confirmed WPDV positive cases in the cited paper by Chang et al.

4. *Line 50 – The sequence of emergent literature is not highly relevant to the reader. The readership would benefit more from an understanding of the current knowledge of the variation across the genomes of the New Zealand and two Australian arteriviruses associated with the disease syndrome described. Understanding the diversity across the three arteriviruses found within wobbly possums seems to be an important basis for interpreting serological responses. A brief summary of the temporal, geographic, and demographic nature of the syndrome in Australia would also benefit the reader and introduce the value of the serological study described.*

Author response: The introduction has been extensively amended and additional information has been added to the revised manuscript.

5. *Line 67: delete different.*

Author response: This change has been made as suggested.

6. *Line 90: perhaps euthanased or died for reasons unrelated to this study (delete naturally from various causes)*

Author response: This change has been made as suggested.

7. *Line 144: Figure 1 and 2 rather than Fig – within the text*

Author response: “Fig” has been used according to the journal specifications, as stipulated in the Manuscript body formatting guidelines (http://www.journals.plos.org/plosone/s/file?id=wjVg/PLOSOne_formatting_sample_main_body.pdf).

8. *Line 164-166: better placed in the materials and methods*

Author response: The map source needs to be retained in the figure caption, as requested by the journal.

9. *Line 170 and elsewhere – univariate seems more universally applied term rather than univariable*

Author response: The statistical analysis has been changed in response to comments from reviewer 2, and the modelling is no longer applicable.

10. *Line 186 – However it is currently unknown which proteins provide the main targets – could be worded in a more natural manner*. *Line 188-89 – may sound more natural by altering the position of the final phrase. Nonetheless, experimentally infected possums showed an increase in corrected OD450 values, using the same antigen, within four weeks….*

### Author response: These sentences have been reworded for clarity (line 227-229, page 11).

### 11. *Line 182 – first line of the discussion describes WPDV as a single entity, when three arteriviruses have been associated with the syndrome and this is important to understand when interpreting likely antigenic similarities and cross reactivity for a test designed for the New Zealand genotype - 70+% genomic divergence*

### Author response: This has been addressed in the introduction (line 49 and 56-64, page 3-4) in the revised manuscript, and the first paragraph in the discussion has been updated to reflect the existence of more than one WPDV.

### 12. *Line 194-196 – The sentence does not make it clear how the findings of prevalence relate to the length of time that the syndrome and disease agents have been in the country. The citations here, do not include the most recent published report by Chang et al, where the temporal distribution of the syndrome is most thoroughly described.*

### Author response: As we outlined earlier, this paper was only available as a pre-print at the time of submission - as this paper has now been published, our revised manuscript (both the introduction and discussion) has been updated to include more discussion of these findings.

### 13. *Line 198 – change has been to were.*

Author response: This change has been made as suggested.

14. *Line 202 – perhaps consider rewording “This is interesting” as it seems a bit presumptive to assume what the reader may find interesting.*

Author response: This has been changed to “this is unexpected”

15. *Line 222-224 – Although samples were collected opportunistically, histological samples could have also been taken from the dead animals examined. Lymphoplasmocytic inflammation often persists through freeze-thaw artefact.*

Author response: A comment has been added to acknowledge histopathology as a useful tool for future studies (line 286, page 14). Unfortunately, as the tissue samples were collected opportunistically, sometimes at various stages of autolysis and underwent several freeze-thaw cycles, histopathology was not considered to be appropriate for the existing sample set.

16. *Line 224 – the language seems awkward and the premise that a single titre could ever tell you when an infection occurred seems flawed*

Author response: This sentence has been removed and replaced with a comment clarifying that paired samples could not be obtained (line 286, page 14).

17. *Line 230 – it is difficult to understand how the reader could interpret the PCR findings without having knowledge of the presence of the three arteriviruses associated with wobbly possum syndrome and the extent of their genomic divergence. It seems very late to be introducing the presence and diversity of the three arteriviruses in line 239-240 – when describing primers*

Author response: The introduction has been extensively updated and introduces what is now known regarding the presence of the three arteriviruses and the genomic divergence. This information was not yet published during preparation of the original manuscript.

18. *Line 240 – delete used*

Author response: This change has been made as suggested.

**Reviewer #2:**

Major comments:

1. *Granted, the statistical approach used here is far better than the usual standard of wildlife publications, which is encouraging. However, the manual backwards stepwise elimination approach is an outdated method for estimating causal effects. A minimum approach should involve each risk factor considered separately in its own model, with covariates added in consideration of causal framework.*

Author response: Many thanks for these comments and for taking the time to review our manuscript.

Having carefully reconsidered the data in light of the above comment, we have decided to remove the multivariable model and pursue a more descriptive, rather than analytical, focus to the present study. We feel that this decision does not take away from the main findings of this study – that is, demonstrated evidence of the widespread circulation of WPDV in Victoria and South Australia, as well as the demonstration of antibodies in two novel possum species. While we agree with the reviewer that the suggested approach would be ideal, the nature of the mixed dataset and the presence of biases that accompany convenience sampling greatly complicate the process of developing a putative causal web accounting for the complexities of this dataset. Additionally, there will also be other important risk factors that cannot be accounted for or for which accurate data cannot be collected in a wildlife survey, unfortunately making any attempted causal framework likely to be incomplete or misinformed.

*The figures should be constructed better for effective communication. A few suggestions:*

2. *for Figure 1, it would be helpful to the reader to present the plots unstacked and with error margins: particularly in view of the relatively small sample sizes, error margins are essential for a reader to readily identify whether or not the seroprevalence differs between the species.*

Author response: Figure 1 has been redesigned in response to this comment. Rather than unstacking the equivocals and seropositives, which we feel would be visually more difficult to read, we have split the figure into two parts. Part A shows the seropositives alone (using the higher, more conservative cut-off point) while part B shows the seropositives and equivocals stacked; both including error bars. This is in line with the way we presented and interpreted the rest of the data in the paper.

3. *Figure 2 is unlikely to print well given its large overall size and the relatively small pie charts; and indeed the use of pie charts is to be avoided (pie charts are a poor medium for comparing outcomes across groups). While a map of source sites is good idea, it could be communicated with a small map of Australia and icons for all sample sources, including north Sydney (omit the pie charts). The comparison of the serological outcome between sample sites could then be presented as a separate figure, in a bar chart with error margins (for the same reasons as Figure 1).*

Author response: This type of figure has been used for similar purposes in previous publications (Legione, Alistair R., et al. "Koala retrovirus genotyping analyses reveal a low prevalence of KoRV-A in Victorian koalas and an association with clinical disease." Journal of medical microbiology 66.2 (2017): 236-244.). However, the figure has been amended for increased clarity, by increasing text sizes and the size of the pie charts.

*Minor comments:*

4. *Lines 29-30: Provide error margins with the proportions.*

Author response: This change has been made as suggested.

5. *Line 39: Suggest replacing ‘Australian brushtail possum’ with ‘common brushtail possum’: it’s best to use the same common name consistently throughout the article. It could also be confusing for readers not familiar with Australian and New Zealand fauna, given the geographical references involved in the sentence too.*

Author response: This change has been made as suggested.

6. *Line 74- 79: Given the age of some of the serum samples, it would be appropriate for brief commentary in the discussion on how that may have affected results in the discussion. It would also be interesting to know amongst relatively old samples, if both seropositives and seronegatives were detected- a sentence or two in the Results would suffice.*

Author response: A sentence has been included in the results section (line 172-174, page 9) to clarify that seropositives, seronegatives and equivocal results were detected in both archival and recently collected samples. The relevant paragraph in the discussion section (line 249-253, page 12) has also been amended to include a comment on this finding, as well as acknowledging possible effect of storage and freeze-thaw cycles on sample quality.

7. *Line 81: Indicate the criteria used to define a possum as being urban, semi-rural or rural.*

Author response: A description of the criteria has been included (line 101-106, page 5-6).

8. *Line 131-132: The approach of modelling the ‘suspect/equivocal’ results in addition to the seropositive results was unusual, but I can understand why given that cut-offs are poorly defined. Some brief, specific commentary in the discussion on the reasoning and the potential implications of misclassification bias would be appropriate. Further, a mention of the relatively small sample size and potential for Type II error influencing study findings is required.*

Author response: The potential for misclassification bias and uncertainty surrounding the cut-offs has been added to the discussion (line 243-246, page 12). We have also presented further discussion on the reasons for potential misclassification bias occurring, given the uncertainty in what cut-off is appropriate to our study. Considering the main research question to demonstrate the presence of WPDV in Australian possums, we have commented further on the strengths of our findings even after adjusting for known test imperfections, and we consider the impacts of any such bias on the main research finding to be negligible.

As we are no longer pursuing a multivariable regression/causal inference, Type II errors are less relevant to the study.

9. *Line 137: It would be helpful to start the Results section with an overall summary of the possum origin of the samples, separately for ELISA and PCR samples. E.g. summarise the numbers of possums by sample source, species, sex, age and environmental origin.*

Author response: The first two sentences of the results section in the revised manuscript describe the possums by origin environment, species, age and sex and point to table 1, where the specific numbers are provided.

10. *Line 173: Table 1: provide error margins for the prevalence estimates.*

Author response: 95% confidence intervals have been added to prevalence estimates in Table 1 and S1.

11. *Line 194-197: Estimates of true prevalence belong in the Results section, with methods for estimation outlined in the Methods section.*

Author response: This has been moved as suggested (to line 170-172 and 160-162, respectively).

12. *Line 199-200: Or more realistically, latent class analysis could be used to estimate prevalence using imperfect tests - identification of ‘known negative’ sera of a poorly understood wildlife infection is not the practical approach. This sentence should be updated accordingly.*

Author response: Bayesian latent class analysis was in fact employed in the original paper [9] – this has now been clarified in the discussion (line 238-239, page 12).

13. *Line 202- 211: To complement this section, a brief comment on known modes of transmission for other arteriviruses in other species would be informative to the reader.*

Author response: Additional information has been added to the manuscript (see page 13, line 264-270).

14. *Line 216-219: Vague commentary like this whilst referencing endangered species is not particularly helpful. How would better understanding the ecology of the virus in Australia and its clinical implications be of value, beyond academic interest? Endemic diseases are not the primary cause of species declines in Australia, so understanding the ecology of the virus and its clinical implications to inform development of interventions to target endemic disease is not a particularly effective or efficient way to aide species recovery. So for what other reason(s) might understanding the ecology of the virus and its clinical implications be helpful for endangered wildlife? Specify clearly or consider omitting this part of the text.*

Author response: An additional brief clarification has been added to the manuscript (line 278-280, page 14). While we agree endemic disease would be unlikely to be the primary cause of species decline, infectious disease can be a known contributing factor in already vulnerable populations. Identifying susceptible species and known reservoirs may help inform on management/intervention strategies such as for example the management of captive breeding colonies (e.g. minimising contact between vulnerable species and common species with high prevalence of infection) or on translocation disease risk analyses (e.g. to avoid introducing the disease to areas in which the virus is absent or, conversely, to prevent translocating susceptible animals to areas in which the virus circulates at a high prevalence).
