# Supplementary material for: Serological evidence for the presence of wobbly possum disease virus in Australia
Source: PLoS One. 2020 Aug 4;15(8):e0237091. doi: 10.1371/journal.pone.0237091 (PMC7402471; doi:10.1371/journal.pone.0237091)
Supplement: Attachment — Submitted filename: Response to Reviewers v3.docx [file pone.0237091.s003.docx]

**Response to reviewers’ comments**

**Reviewer #1:**

The authors have done an excellent job addressing the suggestions of both reviewers. Changes have been thoughtful, clear and concise. The table titles and figures are thoughtfully enhanced. In my opinion the manuscript is acceptable for publication. I offer very minor suggestions, which include:

*Describing the results from the NSW animals - I am certain that there would be interest from the readership*

Authors’ response: Unfortunately only spleen samples were available from NSW (line 89-91). As all of the spleen samples tested in this study were negative for WPDV RNA, the NSW samples are covered under the WPD RT-qPCR results (line 218-221).

*Line 85 – Wild does not need to be capitalised*

*Line 100 – divided – perhaps classified or characterised might read better*

*Line 236 – circulates – to circulate*

*Line 241- The establishment of the cut-offs was (rather than were) hindered*

*Line 267 – although the exact routes of natural transmission have (rather than has) not been fully elucidated*

*Line 316 – found to contain high concentrations (rather than concentration)*

Authors’ response: Thank you for these corrections – we have updated the manuscript to include all of these changes.

**Reviewer #2:**

*Lines 250: “As the authors do not suggest a single optimal cut-off, it is unclear which is most appropriate for our purpose”. This statement comes across poorly, as it vaguely insinuates criticism the validation study for not dictating a one-size-fits-all “optimal cut-off”, which would be entirely inappropriate. The optimal cut-off will depend on the circumstances of the use of the test; specifically, the relative importance of sensitivity and specificity. It is most definitely up to the study authors to decide on the most appropriate cut-off a priori, and consider the ramifications of that choice in interpreting findings. A minor tweak of the wording in view of this would be appropriate.*

Authors’ response: The sentence “As the authors of that prior study do not suggest a single optimal cut-off, it is unclear which is most appropriate for our purpose” has been changed to “In this study, we initially elected to use the more conservative, higher cut-off with a higher specificity and less likelihood of false positive results. However, due to the uncertainty surrounding these cut-off values, the potential for misclassification bias needs to be considered when interpreting the results and, consequently, the results in this study were re-analysed using the lower cut-off for completion.”

*Line 256-257 “our estimate of true seroprevalence of WPDV (22.0%; 95% CI 14.2 – 32.0%) supports the view that this virus has been present and circulating for some time”. This statement is too tenuous and ambiguous; I would recommend citing only the more specific evidence of identification of seropositive individuals amongst samples from 2006.*

Authors’ response: We have removed the speculative suggestion that it has been circulating “for some time” and the sentence has been changed to: “our estimate of the true seroprevalence of WPDV (22.0%; 95% CI 14.2–32.0%) provides strong evidence that the virus is present and circulating in the country”.

*Line 298: matching could be used as part of the design of an investigation into associations between the infection and disease, but it is not essential and so does not deserve specific attention here.*

Authors’ response: We have reworded the sentence slightly to remove the mention of matching and the paragraph now reads: “The design of the current study did not allow associations between WPDV infection and disease to be investigated. This was due to the opportunistic sampling strategy employed, with the majority of samples obtained from diseased or deceased possums and no opportunities for obtaining paired samples to demonstrate rising antibody titres. Collection of tissues for concurrent histological examination could be considered in future studies to look for associated pathology, but these were not available for this sample set”.
